# Supplementary material for: Systematic Review: Targeted Molecular Imaging of Angiogenesis and Its Mediators in Rheumatoid Arthritis
Source: Int J Mol Sci. 2022 Jun 25;23(13):7071. doi: 10.3390/ijms23137071 (PMC9267012; doi:10.3390/ijms23137071)
Supplement: Supplementary file 1 [file ijms-23-07071-s001.zip › Supplementary material_File A_ Specification of RA angiogenesis biomarkers search- March 2022.pdf]

Methods

1. Literature review

This review is reported according to the Preferred Reporting Items for Systematic Reviews and Meta-Analyses (PRISMA) (www.prisma-statement.org).

1.1 Search strategy of RA angiogenesis biomarkers

To identify the relevant publications we conducted systematic searches in the bibliographic databases PubMed, Embase.com and Wiley/Cochrane Library from inception up to March 25, 2022, in collaboration with a medical information specialist.
The following terms were used (including synonyms and closely related words) as index terms or free-text words: "Rheumatoid Arthritis", "Angiogenesis", "Neovascularization", "Markers".
The references of the identified articles were searched for relevant publications. Duplicate articles were excluded. All languages were accepted.

Appendix A / Supplementary material

PubMed Session Results (25 Mar 2022)

Search	Query	Items found
#4	**#1 AND #2 AND #3**	874
#3	**"marker*"[tw] OR "biomarker*"[tw] OR "mediator*"[tw] OR "modulator*"[tiab] OR "stimulator*"[tiab] OR "activator*"[tiab] OR "regulator*"[tiab] OR "signaling"[tiab] OR "signal transduction"[tw] OR "pathway*"[tiab]**	3,679,233
#2	**"Neovascularization, Physiologic"[Mesh] OR "Neovascularization, Pathologic"[Mesh] OR "angiogen*"[tiab] OR "neoangiogen*"[tiab] OR "neo-angiogen*"[tiab] OR "neovascul*"[tiab] OR "neo-vascul*"[tiab] OR "hypervascul*"[tiab] OR "hyper-vascul*"[tiab]**	181,517
#1	**"Arthritis, Rheumatoid"[Mesh:NoExp] OR "rheumatoid arthriti*"[tiab] OR "inflammatory arthriti*"[tiab] OR "autoimmune arthriti*"[tiab]**	147,742

Embase.com Session Results (25 Mar 2022)

Search	Query	Items found
#5	**#4 NOT ('conference abstract'/it OR 'conference review'/it)**	1,326
#4	**#1 AND #2 AND #3**	1,776
#3	**marker*:ab,ti,kw,de OR biomarker*:ab,ti,kw,de OR mediator*:ab,ti,kw,de OR modulator*:ab,ti,kw OR stimulator*:ab,ti,kw OR activator*:ab,ti,kw OR regulator*:ab,ti,kw OR signaling:ab,ti,kw OR 'signal transduction':ab,ti,kw,de OR pathway*:ab,ti,kw**	4,699,444
#2	**'angiogenesis'/exp OR 'neovascularization (pathology)'/de OR angiogen*:ab,ti,kw OR neoangiogen*:ab,ti,kw OR 'neo-angiogen*':ab,ti,kw OR neovascul*:ab,ti,kw OR 'neo-vascul*':ab,ti,kw OR hypervascul*:ab,ti,kw OR 'hyper-vascul*':ab,ti,kw**	269,067
#1	**'rheumatoid arthritis'/de OR 'rheumatoid arthritis synovial fibroblast'/exp OR 'rheumatoid arthriti*':ab,ti,kw OR 'inflammatory arthriti*':ab,ti,kw OR 'autoimmune arthriti*':ab,ti,kw**	246,271

Wiley / Cochrane Library Session Results (25 Mar 2022)

Search	Query	Items found
#4	**#1 AND #2 AND #3**	15
#3	**(marker* OR biomarker* OR mediator* OR modulator* OR stimulator* OR activator* OR regulator* OR signaling OR (signal NEXT transduction) OR pathway*):ab,ti,kw**	136,559
#2	**(angiogen* OR neoangiogen* OR (neo NEXT angiogen*) OR neovascul* OR (neo NEXT vascul*) OR hypervascul* OR (hyper NEXT vascul*)):ab,ti,kw**	8,424
#1	**((rheumatoid NEXT arthriti*) OR (inflammatory NEXT arthriti*) OR (autoimmune NEXT arthriti*)):ab,ti,kw**	17,102
