# Supplementary material for: Systematic Review: Targeted Molecular Imaging of Angiogenesis and Its Mediators in Rheumatoid Arthritis
Source: Int J Mol Sci. 2022 Jun 25;23(13):7071. doi: 10.3390/ijms23137071 (PMC9267012; doi:10.3390/ijms23137071)
Supplement: Supplementary file 1 [file ijms-23-07071-s001.zip › Supplementary material_File B_Specification of RA radiotracers search- March 2022.pdf]

Methods

1. Literature review

This review is reported according to the Preferred Reporting Items for Systematic Reviews and Meta-Analyses (PRISMA) (www.prisma-statement.org).

1.1 Search strategy of RA radiotracers

To identify the relevant publications we conducted systematic searches in the bibliographic databases PubMed, Embase.com and Wiley/Cochrane Library from inception up to March 25, 2022, in collaboration with a medical information specialist.
The following terms were used (including synonyms and closely related words) as index terms or free-text words: "Rheumatoid Arthritis", "Emission-computed Tomography".
The references of the identified articles were searched for relevant publications. Duplicate articles were excluded. All languages were accepted.

PubMed Session Results (25 Mar 2022)

Search	Query	Items found
#3	**#1 AND #2**	1,870
#2	**"Tomography, Emission-Computed"[Mesh] OR "spect"[tiab] OR "petscan*"[tiab] OR "pet"[tiab] OR ("emission"[tiab] AND "tomogra*"[tiab]) OR "positron emission"[tiab] OR "scintigraph*"[tiab] OR "Radiopharmaceuticals" [Pharmacological Action] OR "Radiopharmaceuticals"[Mesh] OR "radiopharmaceutic*"[tiab] OR "radio-pharmaceutic*"[tiab] OR "radiotracer*"[tiab] OR "radio-tracer*"[tiab]**	271,366
#1	**"Arthritis, Rheumatoid"[Mesh:NoExp] OR "rheumatoid arthriti*"[tiab] OR "inflammatory arthriti*"[tiab] OR "autoimmune arthriti*"[tiab] OR "Synovial Membrane"[Mesh] OR "Synoviocytes"[Mesh] OR "synovi*"[tiab]**	187,874

Embase.com Session Results (25 Mar 2022)

Search	Query	Items found
#4	**#3 NOT ('conference abstract'/it OR 'conference review'/it)**	3,491
#3	**#1 AND #2**	4,530
#2	**'computer assisted emission tomography'/exp OR spect:ab,ti,kw OR petscan*:ab,ti,kw OR pet:ab,ti,kw OR (emission NEAR/3 tomogra*):ab,ti,kw OR 'positron emission':ab,ti,kw OR scintigraph*:ab,ti,kw OR 'radiopharmaceutical agent'/exp OR 'radiopharmaceutic*':ab,ti,kw OR 'radio-pharmaceutic*':ab,ti,kw OR 'radiotracer*':ab,ti,kw OR 'radio-tracer*':ab,ti,kw**	555,004
#1	**'rheumatoid arthritis'/de OR 'rheumatoid arthritis synovial fibroblast'/exp OR 'rheumatoid arthriti*':ab,ti,kw OR 'inflammatory arthriti*':ab,ti,kw OR 'autoimmune arthriti*':ab,ti,kw OR 'synovium'/exp OR 'synoviocyte'/exp OR synovi*:ab,ti,kw**	297,769

Wiley / Cochrane Library Session Results (25 Mar 2022)

Search	Query	Items found
#3	**#1 AND #2**	85
#2	**(spect OR petscan* OR pet OR (emission NEAR/3 tomogra*) OR (positron NEXT emission) OR scintigraph* OR radiopharmaceutic* OR (radio NEXT pharmaceutic*) OR radiotracer* OR (radio NEXT tracer*)):ab,ti,kw**	14,244
#1	**((rheumatoid NEXT arthriti*) OR (inflammatory NEXT arthriti*) OR (autoimmune NEXT arthriti*) OR synovi*):ab,ti,kw**	18,810
